# Effect of soluble guanylyl cyclase activator and stimulator therapy on nitroglycerin-induced nitrate tolerance in rats

**DOI:** 10.1186/2050-6511-16-S1-A90

**Published:** 2015-09-02

**Authors:** P Stamm, A Jabs, M Oelze, Y Mikhed, S Kröller-Schön, P Welschof, T Jansen, M Hausding, M Kopp, S Steven, E Schulz, J-P Stasch, T Münzel, A Daiber

**Affiliations:** 1 2nd Medical Clinic, Department of Cardiology, Medical Center of the Johannes Gutenberg University, Mainz, Germany; 2 Bayer Pharma AG, Wuppertal, Germany

## Clinical background

Chronic nitroglycerin (GTN) anti-ischemic therapy induces side effects such as nitrate tolerance and endothelial dysfunction. Both phenomena could be based on a desensitization/oxidation of the soluble guanylyl cyclase (sGC). Therefore, the present study aims at investigating the effects of the therapy with the sGC activator BAY 60-2770 and the sGC stimulator BAY 41-8543 on side effects induced by chronic nitroglycerin treatment. Male Wistar rats were treated with nitroglycerin (100 mg/kg/d for 3.5 days, s.c. in ethanol) and BAY 60-2 770 (0.5 or 2.5 mg/kg/d) or BAY 41-8543 (1 and 5 mg/kg/d) for 6 days.

**Figure 1:**
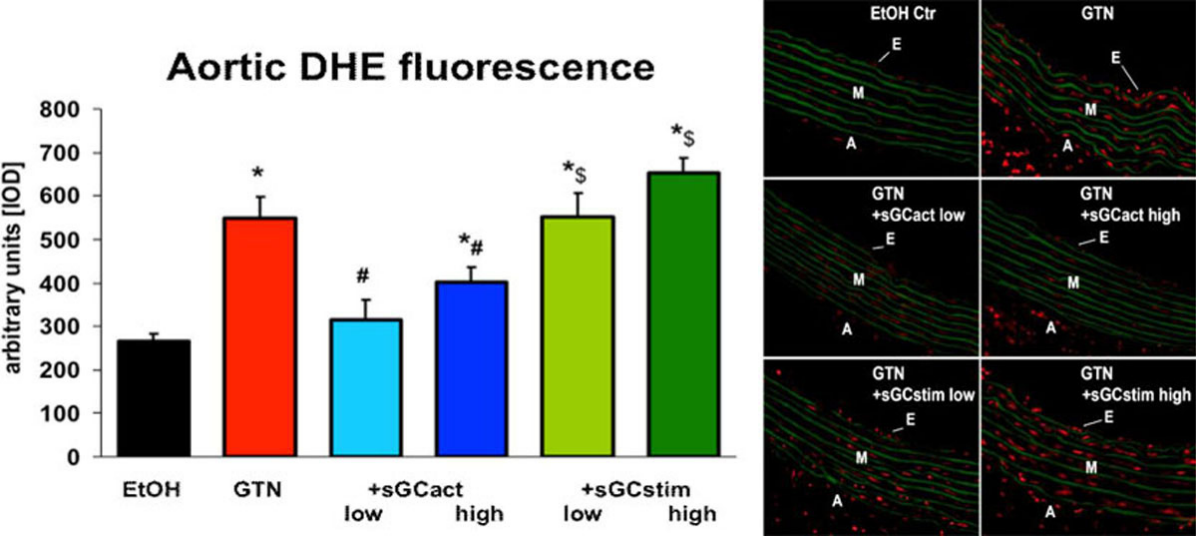
**Aortic DHE fluorescence** Effects of sGC activator BAY 60-2770 and sGC stimulator BAY 41-8543 in vivo treatment on oxidative stress parameters in tolerant rats. Quantification of vascular ROS formation in aortic cryo-sections was assessed by fluorescent microtopography using the superoxide-sensitive dye DHE (1 µM). The data are the means ± SEM from 3-4 animals/group. *, p<0.05 vs. control; ^#^, p<0.05 vs. GTN; ^$^, p<0.05 vs. BAY 60-2770; ^, p<0.05 vs. low dose treatment.

**Figure 2:**
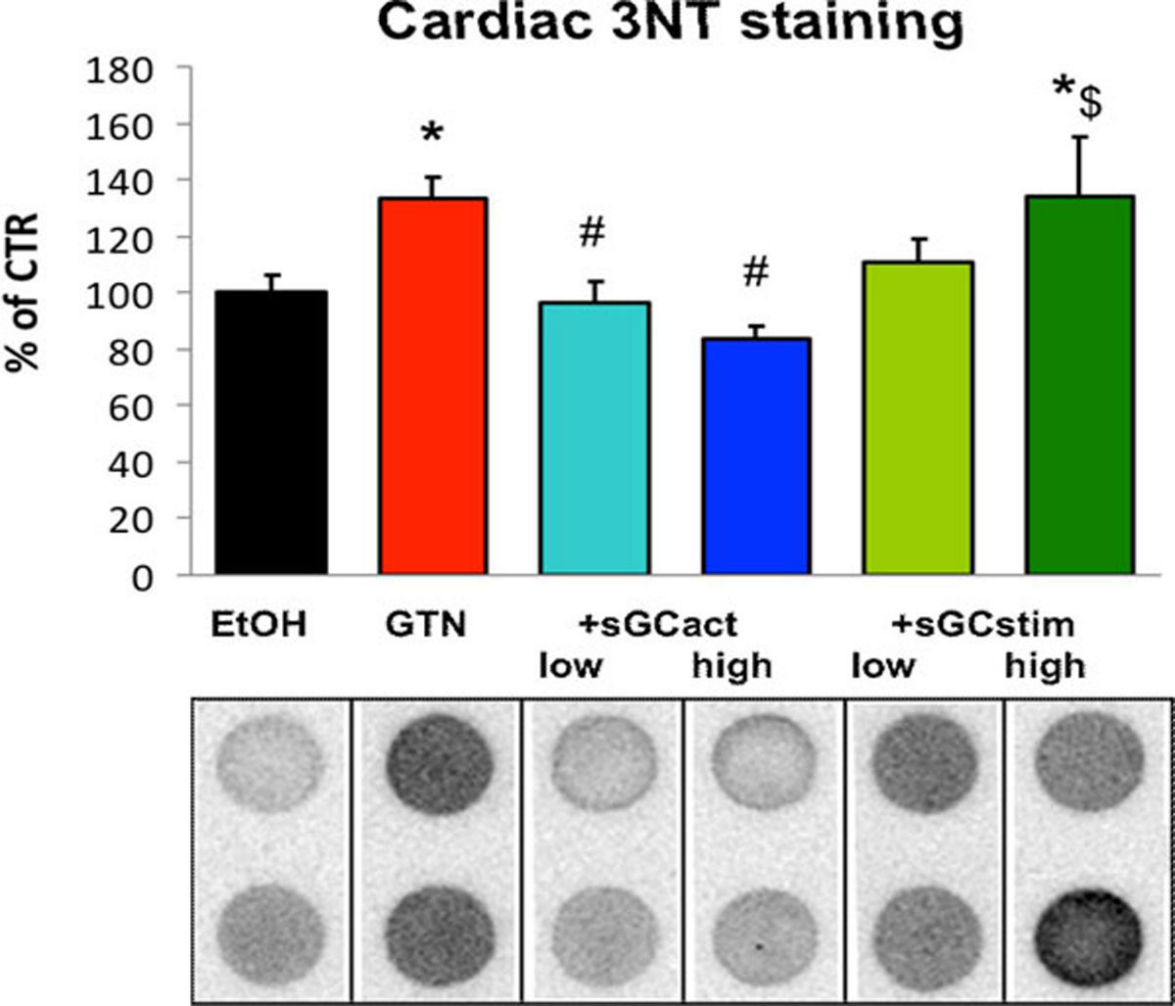
Cardiac 3NT staining

## Conclusion

Therapy with BAY 60-2770 but not with BAY 41-8543 improved nitroglycerin-triggered endothelial dysfunction and nitrate tolerance, corrected the decrease in aortic nitric oxide levels, improved the cGMP dependent activation of protein kinase I in aortic tissue and reduced vascular, cardiac and whole blood oxidative stress (fluorescence and chemiluminescence assays; 3-nitrotyrosine staining). In contrast to BAY 41-8543, the vasodilator potency of BAY 60-2770 was not impaired in isolated aortic ring segments from nitrate tolerant rats. sGC activator therapy improves partially the adverse effects of nitroglycerin therapy whereas sGC stimulation has only minor beneficial effects pointing to a nitroglycerin-dependent sGC oxidation/inactivation mechanism contributing to nitrate tolerance.

